# CdS-Modified TiO_2_ Nanotubes with Heterojunction Structure: A Photoelectrochemical Sensor for Glutathione

**DOI:** 10.3390/nano13010013

**Published:** 2022-12-20

**Authors:** Guo-Na Huo, Sha-Sha Zhang, Yue-Liu Li, Jia-Xing Li, Zhao Yue, Wei-Ping Huang, Shou-Min Zhang, Bao-Lin Zhu

**Affiliations:** 1 College of Chemistry, The Key Laboratory of Advanced Energy Materials Chemistry (Ministry of Education), Tianjin Key Laboratory of Metal and Molecule-Based Material Chemistry, Nankai University, Tianjin 300071, China; 2 Chemistry and Chemical Engineering College, Xingtai University, Xingtai 054000, China; 3 Department of Microelectronics, Nankai University, Tianjin 300350, China; 4 National Demonstration Center for Experimental Chemistry Education (Nankai University), Tianjin 300071, China

**Keywords:** TiO_2_ nanotubes, CdS, heterojunction structure, photoelectrochemical sensor

## Abstract

The formation of heterojunction structures can effectively prevent the recombination of photogenerated electron–hole pairs in semiconductors and result in the enhancement of photoelectric properties. Using TiO_2_ nanotubes (prepared using the hydrothermal-impregnation method) as carriers, CdS-TiO_2_NTs were fabricated as a photoelectrochemical (PEC) sensor, which can be used under visible light and can exhibit good PEC performance due to the existence of the heterojunction structure. The experimental results show that the prepared CdS-TiO_2_NTs electrode had a linear response to 2–16 mM glutathione (GSH). The sensor’s sensitivity and detection limit (LOD) were 102.9 µA·mM^−1^ cm^−2^ and 27.7 µM, respectively. Moreover, the biosensor had good stability, indicating the potential application of this kind of heterojunction PEC biosensor.

## 1. Introduction

Glutathione (GSH) is a free-radical scavenger in the body and plays a primary role in functions such as immune regulation, gene expression regulation, enzyme activity, etc. [1]. Many human diseases can influence GSH concentration, and GSH detection is very important due to its significance in physiological circumstances [2]. Among different biomolecule detection methods, photoelectrochemical (PEC) detection is prominent due to its high response speed, high sensitivity, and simple instruments [3].

PEC sensors’ foundations are based on the relation between concentrations of the detected substances and photocurrents. The PEC sensor, which is commonly fabricated by photoactive materials, is firstly photoexcitated to form electron–hole pairs [4,5,6]. During the reaction of photoexcitation species with analytes, the photocurrent is obtained. Thus, a crucial aspect of a prominent PEC sensor is the selecting of an excellent photoelectrode, which will control the sensor’s sensitivity, detection limit (LOD), etc. [7].

Nowadays, the electrode materials with photoelectric responses are focused on inorganic materials, such as TiO_2_ [8], CdS [9,10], ZnO [11], and CuO [12]. Among them, TiO_2_ is most prominent due to its stable chemical properties, high PEC performance, and good biocompatibility [13]. Compared with common powder materials, TiO_2_ nanotubes are more prominent for their unique 1D nanostructure, which can shorten the distance between photogenerated carriers and accelerate electron transport [14]. The modifiers and aimed samples can also be highly dispersed across the nanotubes, and result in a quick response. As is common sense, UV rays are harmful to most biological molecules. Due to its big band gap, the pure TiO_2_ photoelectrode can only be used under UV light, which limits the utility of TiO_2_ as a biosensor [15]. The fast recombination of photogenerated charges is another shortcoming of TiO_2_, which decreases the PEC sensor’s sensitivity.

To improve the PEC performance of TiO_2_, such as narrowing its energy band and improving its electron-hole separation efficiency, different metals and nonmetals (C [16], S [17], N [18], Fe [19], Ti [20], Bi [21], and so on) were used as dopants, and precious metals (Au [22], Pt [23,24], Ag [25], and so on) were used as modifications. Doping and modification can decrease the recombination ratio of photoelectrons and photoholes and promote their separation in TiO_2_ [26,27]. However, the impurity level formed by element doping may become the recombination site of the photogenerated electrons and holes, and precious metals are expensive and easily lost in the PEC reaction process.

As has been generally reported, constructing heterojunctions with other semiconductors (CdS [8], CuO [28], MoS_2_ [29], g-C_3_N_4_ [30], SnS_2_ [31], and so on) is also an effective way to improve TiO_2_’s PEC performance. Among the above-mentioned semiconductors, CdS’s band gap is 2.3 eV, which can lead to a higher absorption coefficient under visible-light illumination. However, the unstable properties of CdS limits its practical utility. Forming composite materials with stable TiO_2_ is a good solution to this limitation. In our previous work, we have prepared TiO_2_ nanotube-supported CdS as a photocatalyst. Compared with pure TiO_2_ nanotubes or CdS nanoparticles, the fabricated composite exhibited better photoelectric properties [32]. According to the simulation of density functional theory (DFT), the formation of the heterojunction structure can also be considered the existence of an internal electric field [33]. After the heterojunction structure is formed in the CdS-TiO_2_ system, the charge-transfer barrier is decreased, and the photogenerated electrons and holes can be effectively separated, resulting in a faster charge transport rate and lower recombination efficiency. Therefore, the formed CdS-TiO_2_ sensors are not only active under visible light but can also lead to better sensing properties being obtained for biomolecules [34,35]. It has been reported that CdS modification can obtain 2.9-fold PEC signal enhancement compared with that of 0.1% for Fe-TiO_2_ [36].

Based on the improved PEC properties of the heterojunction structure, a TiO_2_ nanotube was modified with powdery CdS to form a CdS-TiO_2_NTs electrode, which was further applied for GSH detection in this paper. Through the characterization, investigation of PEC sensitivity, and stability of the CdS-TiO_2_NTs electrode, the practicality and sensing mechanism of the fabricated CdS-TiO_2_NTs sensor is also discussed.

## 2. Experiment

### 2.1. Synthesis of CdS-TiO_2_NTs

Hydrogen-titanate nanotubes prepared using the hydrothermal method were soaked in titanium sol for 10 min, naturally dried in the air, and calcined at 400 °C for 2 h to obtain TiO_2_NTs [37]. A certain amount of sulfur powder was dissolved in tetrahydrofuran solution. After a quantity of Cd(NO_3_)_2_·4H_2_O was slowly added through stirring, the prepared TiO_2_NTs were added. The calculated CdS/TiO_2_ molar ratio was 1:1. NaBH_4_ solution was slowly added under the condition of an ice water bath and N_2_. After being stirred for 30 min, the solution was ultrasonic treated for 30 min, and then stirred overnight. The samples were successively cleaned by tetrahydrofuran, water, and ethanol, and then treated in a vacuum drying oven to obtain CdS-TiO_2_NTs.

### 2.2. Preparation of CdS-TiO_2_NTs Electrode

20 µL of CdS-TiO_2_NTs solution was dropped on a glassy-carbon electrode. After the dropped solution was dried, the rest of the solution was added, and 15 µL of 3% chitosan solution was dropped. After naturally air drying overnight, the CdS-TiO_2_NTs electrode was obtained.

### 2.3. Photoelectrochemical Testing

The CdS-TiO_2_NTs electrode’s electrochemical properties were investigated using the three-electrode system from the Zahner electrochemical workstation. The CdS-TiO_2_NTs electrode, Ag/AgCl electrode, and Pt electrode were used as the working electrode, reference electrode, and auxiliary electrode, respectively. An LED lamp with visible light (429 nm, 35 W/m^2^) was used as the illumination source, and the pH of electrolytes was maintained at 7.4.

### 2.4. Instrument Model

XRD patterns were obtained using the Rigaku D/MAX-2500 X-ray diffractometer (Rigaku SmartLab, Rigaku Corporation, Tokyo, Japan). Scanning electron microscope (SEM) and energy dispersive spectrometer (EDS) images were observed on JSM-7800F (Japanese electronics, Beijing, China). To obtain TEM and HR-TEM images, JEM-2800 (Japanese electronics, Shanghai, China) and the Talos F200X G2 transmission electron microscope were used, respectively. X-ray photoelectron spectroscopy (Kratos Axis Ultra DLD (Kratos Analytical Ltd., Manchester, UK), Al target, double anode Al/Mg target) recorded the XPS patterns. All the electrochemical experiments were performed on the Zennium electrochemical workstation (Kronach–Gundelsdorf, Germany, bias voltage: 0.5 V).

## 3. Results and Discussion

### 3.1. Surface Morphology Characterization

**Figure 1:**
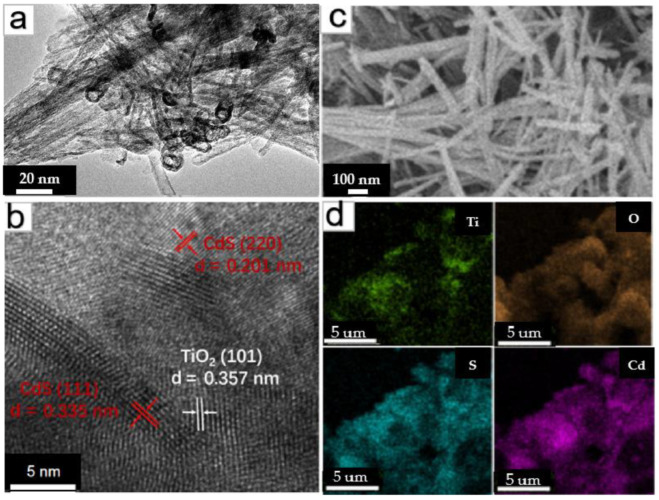
TEM (**a**), HR-TEM (**b**), and SEM (**c**) images of the CdS-TiO_2_NTs and mapping (**d**) of CdS-TiO_2_NTs.

**Figure 2:**
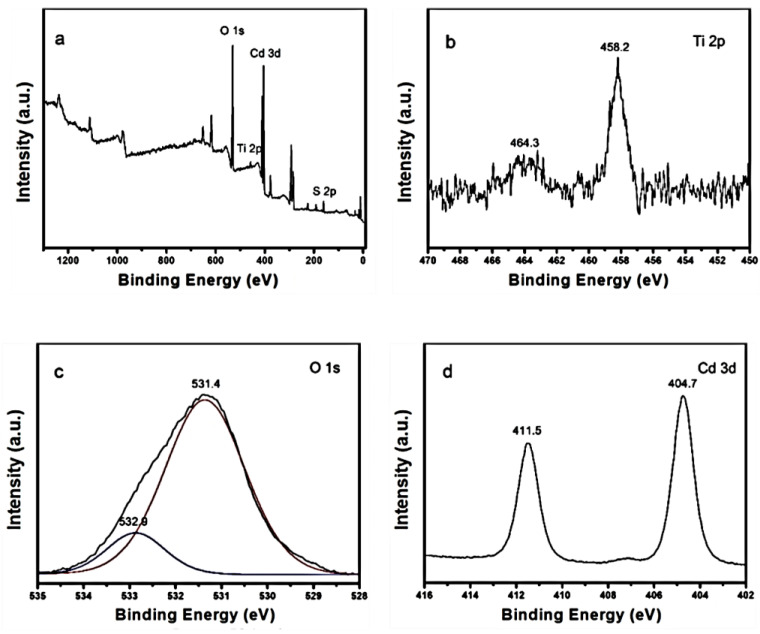

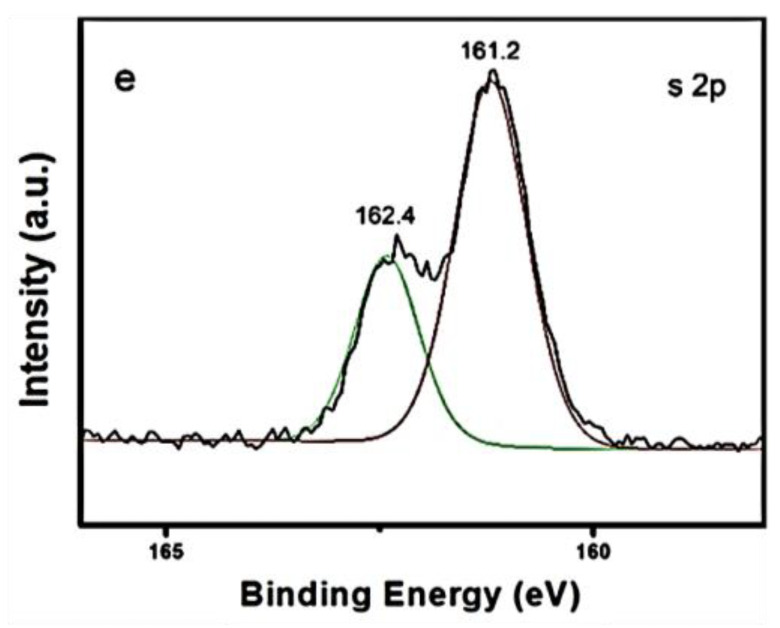
(**a**) Survey XPS spectrum of the CdS-TiO_2_NTs and high-resolution spectra for (**b**) Ti 2p, (**c**) O 1s, (**d**) Cd 3d, and (**e**) S 2p.

Figure 1a,b shows the TEM and high-resolution TEM images of CdS-TiO_2_NTs. From Figure 1a, it can be clearly seen that the open-ended nanotubes have a diameter of about 10 nm. Compared with the pure TiO_2_NTs, there are many substances with dark color and ring shapes in the tubular materials, which should be the shadow of heavy metal cadmium. In Figure 2b, the measured lattice spacings of 0.335 nm, 0.201 nm, and 0.357 nm, correspond to the (111) and (220) planes of CdS and the (101) plane of anatase TiO_2_, respectively. It demonstrates the coexistence and association of CdS and TiO_2_NTs and the formation of the heterojunction structure. To verify the existence of Cd and S more directly, SEM and corresponding mapping images (Figure 1c,d) of the CdS-TiO_2_NTs were obtained. It is evident that the TiO_2_NTs are modified by cadmium and sulfide, and the atomic ratio of Cd/S is close to 1:1, corresponding to the composition of CdS. Some regions in Figure 1d exhibit different element contents. This could be attributed to the lack of a uniform distribution in the components of CdS-TiO_2_NTs.

XPS measurement was used to characterize the chemical composition of the CdS-TiO_2_NTs. Figure 2 shows the full XPS spectrum (Figure 2a), and Ti 2p, O 1s, Cd 3d, and S 2p high-solution spectra (Figure 2b–e) of CdS-TiO_2_NTs. As shown in Figure 2a, the CdS-TiO_2_NTs sample contained chemical elements of O, Ti, Cd, and S. Two Ti 2p peaks emerged at 458.2 eV and 464.3 eV in the CdS-TiO_2_NTs that corresponded to Ti 2p_1/2_ and Ti 2p_3/2_ (Figure 2b), indicating the chemical state Ti^4+^ of titanium oxide. As displayed in Figure 2c, the O 1s XPS spectrum showed two peaks, with the one at 531.4 eV corresponding to lattice oxygen, and the other at 532.9eV corresponding to adsorbed oxygen. As shown in Figure 2d, the peaks at BE of 404.7 eV and 411.5 eV corresponded to Cd 3d_5/2_ and Cd 3d_3/2_, respectively, indicating the existence of Cd^2+^ in CdS-TiO_2_NTs. The high-solution S 2p peaked at 161.2 eV and 162.4 eV, which is evidence for S^2-^ in CdS. In summary, the XPS results in Figure 2a–e show the coexistence of CdS and TiO_2_, consistent with the results of TEM and SEM.

**Figure 3:**
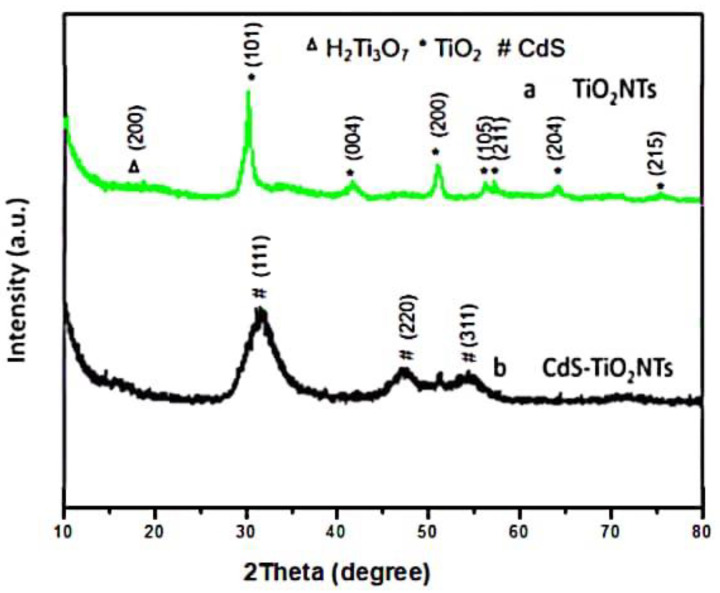
XRD patterns of TiO_2_NTs (a) and CdS-TiO_2_NTs (b).

In order to investigate the composition of the prepared materials, XRD patterns were performed. Figure 3 shows the XRD patterns of TiO_2_NTs and CdS-TiO_2_NTs. Most of the peaks on pattern a in Figure 3 corresponded to that of anatase TiO_2_ (JCPDS# 21-1271), indicating that the TiO_2_NTs are mainly anatase type. The peak at 11° corresponded to the (200) crystal plane of H_2_Ti_3_O_7_ (JCPDS # 47-0561), which is the undecomposed hydrogen titanate. As shown on pattern b in Figure 3, CdS diffraction peaks (JCPDS# 10-0454) emerged in CdS-TiO_2_NTs. The addition of CdS weakened the diffraction peaks of TiO_2_, which could be attributed to the high concentration of CdS in TiO_2_. Moreover, the wide diffraction peaks indicate the small particle sizes of CdS and TiO_2_. By combining the results of the above TEM, SEM, and XPS analyses, the successful loading of CdS on TiO_2_ nanotubes can be confirmed.

**Figure 4:**
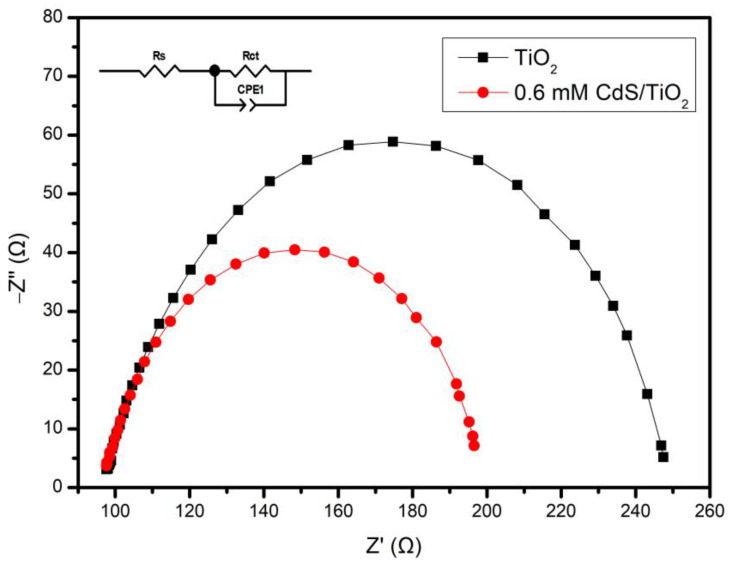
EIS of TiO_2_ and CdS/TiO_2_NTs.

EIS is commonly used to detect electrode–electrolyte interactions and charge-transfer phenomena [38]. The arc radius in the EIS Nyquist plot reveals the charge-transfer resistance, and charge-transfer ability of the electrode [39]. As shown in Figure 4, the Rs of the samples were almost all the same. However, the Rct for the TiO_2_NTs and CdS/TiO_2_NTs were 153 Ω and 102.7 Ω, respectively. Compared with TiO_2_NTs, the value of the CdS/TiO_2_NTs was smaller. This could be attributed to the formation of a heterojunction structure in the CdS/TiO_2_NTs, which resulted in the rapid transmission of charges and reduced charge-transfer resistance. Under light illumination, the effective separation and transmission of photogenerated carriers resulted in the small Rct of CdS/TiO_2_NTs. In the PEC process, the small Rct can facilitate the transfer of electrons and result in a high photocurrent.

### 3.2. Electrochemical Characterization

**Figure 5:**
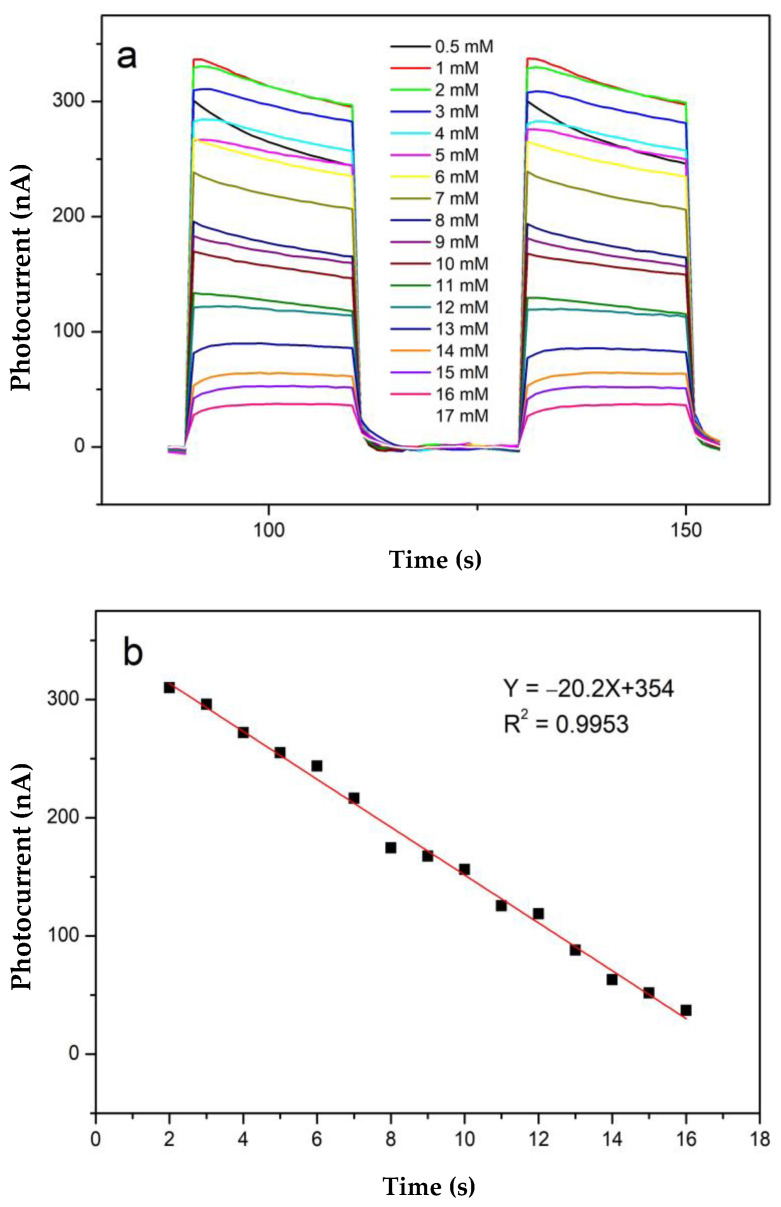
(**a**) Photocurrent response for the detection of different concentrations of GSH (2–16 mM), and (**b**) corresponding photocurrent-GSH concentration curve of the CdS-TiO_2_NTs under irradiation of 429 nm.

The sensitivity of the fabricated CdS-TiO_2_NTs for GSH was monitored under 429 nm LED-light irradiation. Figure 5a shows the photocurrent–time curves of the CdS-TiO_2_NTs in GSH solutions with 18 different concentrations, which ranged from 0.5 mM to 17 mM. Figure 5b displays the relationship between the GSH concentrations and the photocurrents of the CdS-TiO_2_NTs. The photocurrent-GSH concentration showed a linear relationship in the range of 2–16 mM. The regression equation is Y = −20.2X + 354 (R^2^ = 0.9953) (Y referring to photocurrent, and X referring to GSH concentration). The detection limit (LOD) of the CdS-TiO_2_NTs biosensor was calculated based on the equation of LOD = 3 Sb/S (Sb is the standard deviation of the blank signal, and S is the sensitivity of the fabricated sensor), which was equal to 27.7 µM.

**Figure 6:**
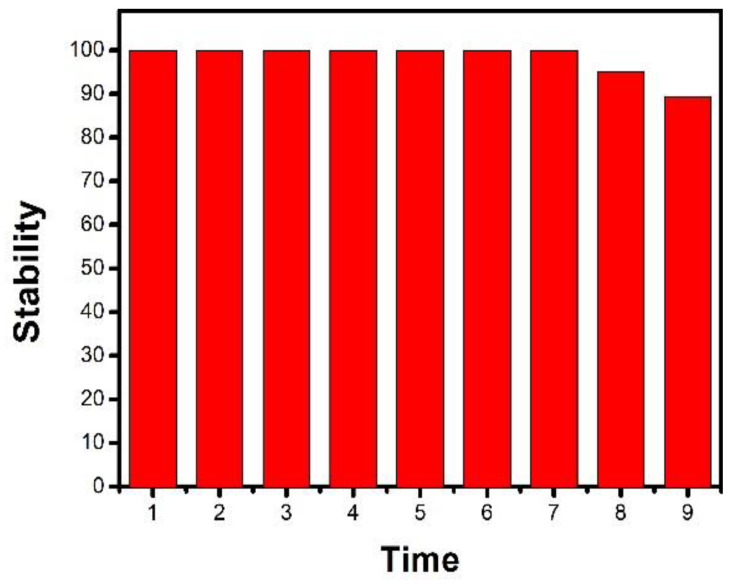
Relationship between time and response of the CdS-TiO_2_NTs electrode.

The stability of the CdS-TiO_2_NTs sensor was also investigated. The CdS-TiO_2_NTs electrode was stored in air at room temperature. Its current response to PBS solution was monitored 9 times under 429 nm LED-light irradiation (bias voltage 0.3 V). As shown in Figure 6, the CdS-TiO_2_NTs maintained at least 89.3% of the initial value during this test, suggesting the relatively high stability of the fabricated CdS-TiO_2_NTs electrode [39].

TiO_2_NTs exhibit no sensitivity to GSH under visible light. However, the CdS-TiO_2_NTs presented good sensitivity under the same condition. Obviously, the modification of CdS endowed the TiO_2_ with PEC activity under visible light. Moreover, the heterojunction structure of the CdS-TiO_2_ composite induced rapid transmission of the charges. In our previous work, a density functional theory (DFT) simulation was conducted for a similar CdS-TiO_2_ composite [40]. As indicated by the simulation results, tunnels formed at the interlayer section of the CdS-TiO_2_ composite and facilitated the charge transfer between CdS and TiO_2_. During the PEC process, CdS was firstly excited under visible light. Because the conduction bands (CBs) of CdS are higher than that of TiO_2_, the excited electrons in CdS transferred to the CBs of TiO_2_ and generated a photocurrent.

**Figure 7:**
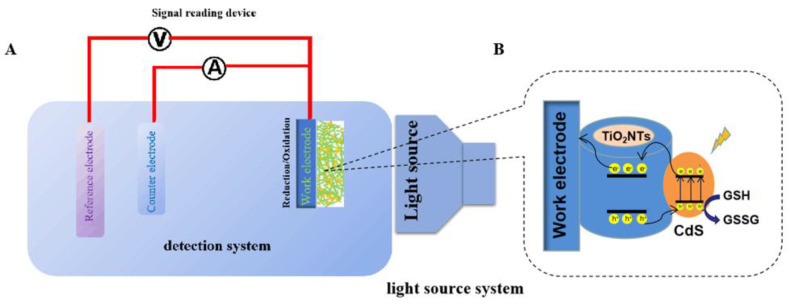
Schematic illustration of the PEC process for GSH detection by the CdS-TiO_2_NTs electrode biosensor ((**A**) is the detection system and (**B**) is the work electrode).

Based on the experiment results and our former simulation results, the PEC detection process for GSH by CdS-TiO_2_NTs electrode biosensor in this work was preliminarily put forward to explain the improved performance of CdS-TiO_2_NTs, as shown in Figure 7. Under the illumination of visible light, electrons are photo-generated in CdS and transferred from the CBs of CdS to the CBs of TiO_2_, reaching the glassy carbon electrode and eventually the counter electrode. Meanwhile, the holes in CdS react with GSH to form GSSG. During this process, the inner self-combination of electrons and holes in both CdS and TiO_2_ are decreased, and electrons and holes are accelerated at different parts. Thus, the association of CdS with TiO_2_ can result in superior PEC performance.

## 4. Conclusions

In summary, CdS-TiO_2_NTs with a heterojunction structure were successfully designed as a PEC sensor for GSH detection. The CdS-TiO_2_NTs exhibited good PEC properties due to the effective charge transfer between CdS and TiO_2_. The photocurrent signals of the CdS-TiO_2_NTs biosensor were linear to 2–16 mM GSH solution. In addition to GSH, future applications of this PEC sensor to other substances are anticipated.

## Data Availability

The data presented in this study are available on request from the corresponding author.
